# Machine Learning Prediction Models for Chronic Kidney Disease Using National Health Insurance Claim Data in Taiwan

**DOI:** 10.3390/healthcare9050546

**Published:** 2021-05-07

**Authors:** Surya Krishnamurthy, Kapeleshh KS, Erik Dovgan, Mitja Luštrek, Barbara Gradišek Piletič, Kathiravan Srinivasan, Yu-Chuan (Jack) Li, Anton Gradišek, Shabbir Syed-Abdul

**Affiliations:** 1 School of Information Technology and Engineering, Vellore Institute of Technology (VIT), Vellore 632014, India; surya.thiru001@gmail.com (S.K.); kathiravan.srinivasan@vit.ac.in (K.S.); 2 Department of Biotechnology, Indian Institute of Technology Madras, Chennai 600036, India; kapeleshh@gmail.com; 3 Department of Intelligent Systems, Jozef Stefan Institute, Jamova Cesta 39, 1000 Ljubljana, Slovenia; erik.dovgan@ijs.si (E.D.); mitja.lustrek@ijs.si (M.L.); 4 Novo Mesto General Hospital, Šmihelska Cesta 1, 8000 Novo Mesto, Slovenia; barbara.gradisek@gmail.com; 5 Graduate Institute of Biomedical Informatics, College of Medical Science and Technology, Taipei Medical University, Taipei 110, Taiwan; jack@tmu.edu.tw

**Keywords:** chronic kidney disease, deep learning, machine learning, electronic health records

## Abstract

Chronic kidney disease (CKD) represents a heavy burden on the healthcare system because of the increasing number of patients, high risk of progression to end-stage renal disease, and poor prognosis of morbidity and mortality. The aim of this study is to develop a machine-learning model that uses the comorbidity and medication data obtained from Taiwan’s National Health Insurance Research Database to forecast the occurrence of CKD within the next 6 or 12 months before its onset, and hence its prevalence in the population. A total of 18,000 people with CKD and 72,000 people without CKD diagnosis were selected using propensity score matching. Their demographic, medication and comorbidity data from their respective two-year observation period were used to build a predictive model. Among the approaches investigated, the Convolutional Neural Networks (CNN) model performed best with a test set AUROC of 0.957 and 0.954 for the 6-month and 12-month predictions, respectively. The most prominent predictors in the tree-based models were identified, including diabetes mellitus, age, gout, and medications such as sulfonamides and angiotensins. The model proposed in this study could be a useful tool for policymakers in predicting the trends of CKD in the population. The models can allow close monitoring of people at risk, early detection of CKD, better allocation of resources, and patient-centric management.

## 1. Introduction

Chronic Kidney Disease (CKD) is a condition resulting in insufficient kidney function, where patients have to live with a compromised quality of life. Asia has the highest prevalence of CKD in the world, led by Japan and followed by Taiwan. In Taiwan, CKD has been the eighth leading cause of death since 1997. Compared to other countries, Taiwan has higher incidences and mortality rates, with the prevalence increasing from 1.99% in 1996 to 9.83% in 2003 [1], while awareness about CKD has remained low [2].

CKD is a substantial financial burden on patients, healthcare services, and the government. Treatments of the ESRD with Renal Replacement Therapy are either expensive (hemodialysis and peritoneal dialysis) or complex (transplantation). Taiwan has about 0.1%–0.2% of the population receiving dialysis—contributing to about 7% of the total budget of the National Health Insurance (NHI) program [3]. The association of CKD with other chronic diseases also exacerbates the situation. From the public health perspective, it is therefore imperative to be able to predict the trends in terms of CKD prevalence so that timely decisions can be taken by the decision-makers (ministries, insurers, hospital managers, etc.) to mitigate a potential surge in the number of cases. Such mitigation measures can include enhanced population screening for CKD-related risks and awareness campaigns, as it has been demonstrated that lifestyle changes (reducing body weight, improving diet, increasing physical activity, reducing alcohol consumption, avoiding smoking, early referral to nephrologists, proper use of medication, and treatments to control other risk factors) are the most effective measures to combat the exacerbation of the condition with minimal associated costs [4]. Further mitigation strategies are setting up enough facilities for hemodialysis and training the personnel.

With the availability of biomedical data, the use of machine-learning techniques in healthcare for developing disease prediction models has become common. Further, methods such as deep learning and techniques like ensemble learning have greatly improved the predictive power of machine learning models. By deriving features from Electronic Health Records (EHR), accurate disease prediction models can be developed [5,6]. At the patient level, a physician can assess the onset of CKD using laboratory tests by looking at standard parameters such as the glomerular filtration rate (eGFR) and the albumin-creatinine ratio [7]. On the other hand, from the public health perspective, laboratory data is typically not available on a large scale. However, two types of data can generally be extracted from the insurance companies’ databases: diagnoses and medications for each patient’s visit at the hospital.

Common approaches for developing disease prediction models with EHR data involve collecting clinical and laboratory data from sources such as billing or claims data, discharge summaries, patient history, etc., and building models on features extracted from them. Some previous studies attempted to use longitudinal data to capture temporal patterns to develop disease prediction models for CKD. Ren et al. (2019) developed a predictive model for kidney disease among patients with hypertension from EHR consisting of textual and numeric information. They proposed a neural network framework based on Bidirectional long short-term memory and auto-encoders to encode the textual and numerical information, respectively. They performed under-sampling to balance the data. They achieved 89.7% accuracy with 10-fold cross-validation [8]. Song et al. (2020) presented a one-year prediction model using a landmark-boosting approach based on gradient boosting machines for CKD among diabetes patients with an AUROC of 0.83. They analyzed longitudinal data containing several clinical observations derived from EHR and billing data [9]. Fenglong et al. (2018) proposed a general framework using posterior regularisation techniques that incorporate prior medical information from the EHR for prediction models. The constraint feature design in the framework took into consideration patient characteristics, underlying disease, disease duration, genetics, and family history. The patient characteristics included sex, age, and ethnicity. While using prediction models for a certain disease, the framework took into consideration the diagnosis of the underlying disease that would be related to the occurrence of the main disease to be predicted [10]. Another similar work by Katsuki et al. (2018) predicted the risk of entering the second stage diabetic nephropathy from the first stage using EHR data consisting of sequences of lab test results. They used convolutional autoencoders to encode the temporal features and achieve performance better than baseline models [11].

Similarly, some studies used non-temporal EHR data to develop disease prediction models. Song et al. (2019) extracted several significant clinical features from EHR data using an ensemble feature selection method to predict the risk of kidney disease among diabetes patients. They achieved an AUROC of 0.71 on an external validation set [12]. Jardin et al. (2012) predicted kidney-related outcomes among diabetes patients using the Cox proportional hazard regression on the ADVANCE cohort, which comprises demographic, behavioral, and physical information, and relevant lab values. They achieved a C statistics of 0.847 on predicting major renal events [13]. Dovgan et al. (2020) predicted the onset of renal replacement therapy three, six, and 12 months after the CKD diagnosis. For their 12-month prediction, they achieved an AUC of 0.773 [14].

In this paper, we aimed to develop machine-learning models that predict the onset of CKD within the next 6 and 12 months. The model is based on the insurance claims data (age, sex, comorbidities, and medication) over an observation period of 24 months. Further, we aim to assess the reliability of the models by identifying the comorbidities and medications that impact the development of CKD the most.

## 2. Materials and Methods

### 2.1. Study Design

**Figure 1:**
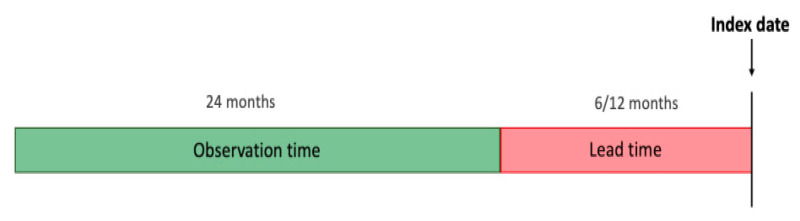
Chronology of time periods and events.

This is a retrospective study on patients who have been diagnosed with CKD and a group of patients without CKD within the chosen observation period. We define the **index date** as the time at which the patient was diagnosed with CKD (ICD9 code: 585, 586) for the first time. For the non-CKD group, the index date is randomly sampled while ensuring a similar distribution of years with the CKD group. We aim to predict the onset of CKD 6 and 12 months before the index date (referred to as the **lead time**) by processing data from the preceding two years from the lead time (referred to as the **observation time**). Figure 1 illustrates the chronology of time periods and events.

### 2.2. Dataset

**Figure 2:**
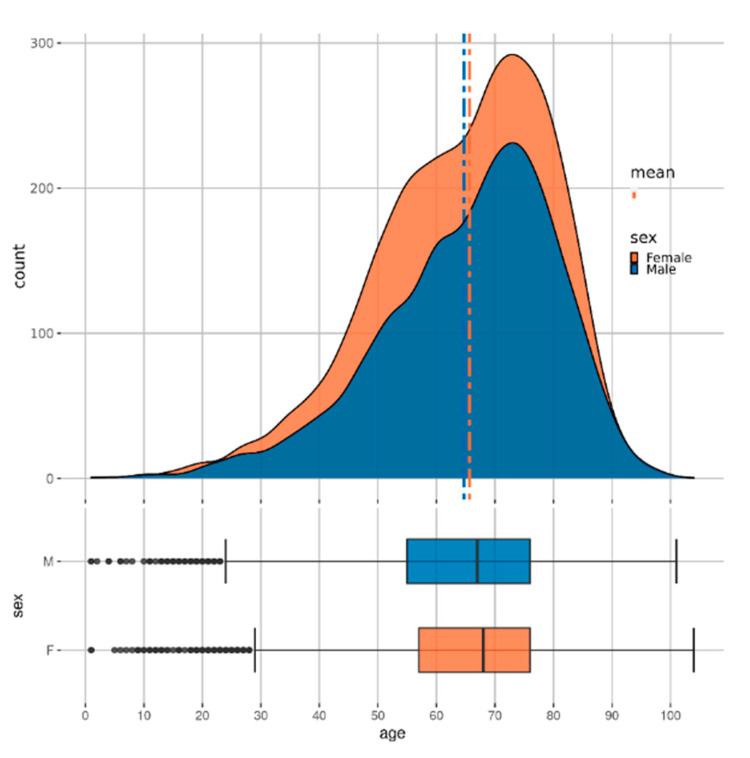
Distribution of age and sex in the CKD cohort.

The study was conducted using Taiwan’s National Health Insurance Research Database (NHIRD) [15], comprising patients’ insurance claims data from 1997 to 2012. Each patient record consists of a patient’s comorbidity or drug prescription by date. The comorbidities are represented by their ICD 9 codes and medications by their ATC codes. Figure 2 shows the distribution of patients with CKD across age and sex. Some further analysis of the dataset is available in the Supplementary Materials.

### 2.3. Data Processing

**Figure 3:**
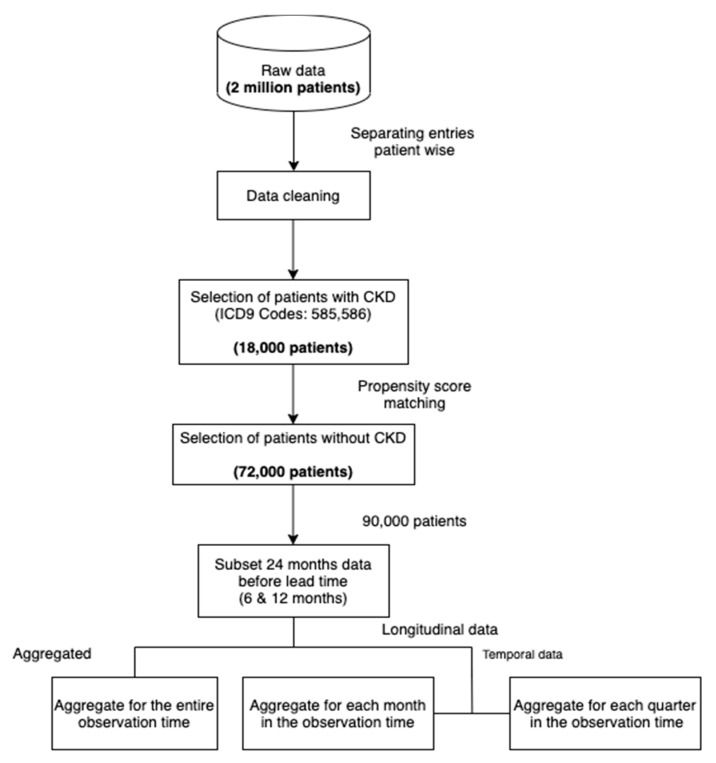
Data processing pipeline.

Figure 3 shows our data processing pipeline. The data cleaning step involved dropping duplicates, missing and incorrect values. In addition, we only included patients of ages below 100. Table 1 shows the primary characteristics of the dataset.

**Figure 4:**
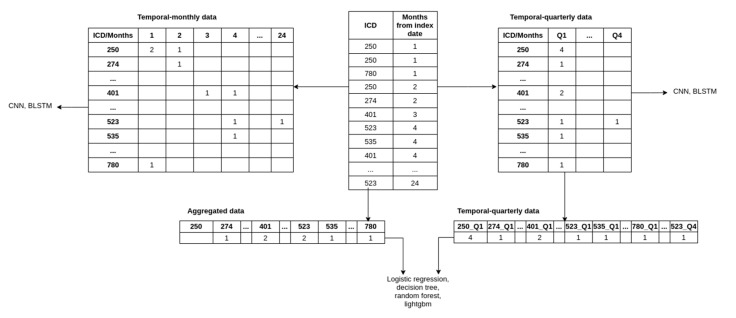
Example of temporal, temporal-quarterly, and aggregated data prepared from the raw data.

To subsample a population for our study, we selected all the patients with at least 2 recorded CKD diagnoses across visits (approximately 18,000). We then sampled non-CKD patients (approximately 72,000) using propensity score matching [16] with the variables age and sex. In this method, the probability of developing CKD, conditional on the covariates (age and sex), is obtained for each sample using a logistic regression model (this probability is called the propensity score). For each case of CKD, the method then finds four non-CKD cases from the set of all non-CKD individuals with the propensity scores closest to the propensity score of the CKD case. By doing so, we ensure the same distribution of the variables age and sex in both subsets (CKD and non-CKD). Such a selection of data reduces the selection bias and leads to a better causal inference [17]. As diseases like CKD often have a high correlation with age (see Figure 2), performing the matching can lead to more reliable and meaningful results than random sampling. The ratio of 1:4 was chosen as a trade-off between machine learning algorithms preferring balanced data and the higher number of non-CKD patients. The final step consisted of structuring the raw data into a dataset appropriate for machine learning, as demonstrated in Figure 4. More precisely, we produced three datasets that included:

1. Aggregated data;
2. Temporal monthly;
3. Temporal quarterly.

To obtain the aggregated data, we discarded the temporal component by summing the occurrences of comorbidities and medications for each patient across the observation period. The aggregated data is thus represented with a vector where each element represents the count of comorbidities/medications throughout the observation period. Including age, sex, comorbidities, and medications, the processed data contained 1504 features.

The temporal-monthly was obtained by aggregating the comorbidities and medications over each month during the observation period. Each patient’s comorbidities and medications were thus represented with a matrix where each row represents the comorbidities (ICD codes) or medications (ATC codes), and each column represents the month of observation (i.e., months from the beginning of the lead time). The index (i, j) in the matrix represents the number of times the patient was diagnosed with/prescribed the i^th^ comorbidities/medication during the j^th^ month from the end of the observation period.

To obtain the temporal-quarterly data, we aggregated the features across each quarter (a total of 8 quarters for our data) and created a vector and a matrix (as described above) of input for each patient. Because the flattened vectors create an intractable number of features, we limited the number of features by taking the top 100 comorbidities/medications features through feature selection (using the LightGBM model, which is described later in the text). Adding age and sex to the feature set results in 802 features for each patient. We used the same feature set for the matrix variant to ensure comparability.

Note that the vector variant of the temporal-quarterly data and aggregated data was in a one-dimensional vector because it was used with classical machine learning, and this is the required type of input for such algorithms. The matrix variant of the temporal-quarterly and temporal-monthly data was in a two-dimensional matrix and was used with our deep neural network architectures, for which the two-dimensional data was appropriate.

### 2.4. Model Development

**Figure 5:**
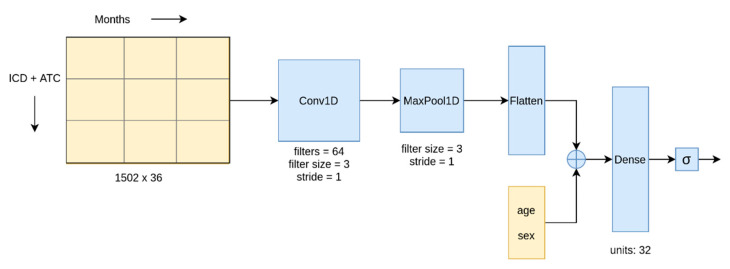
The CNN architecture used in this study.

We used various modeling algorithms from packages like Scikit-learn [18] (Logistic regression, decision tree, random forest [19,20,21]), LightGBM [22], and TensorFlow (Convolutional neural networks, Bi-directional long short term memory) [23] on the preprocessed dataset. We used a 1D Convolution layer, which can perform convolution across a temporal dimension. Figure 5 shows our CNN architecture. In the BLSTM network, the convolution layer in the architecture was replaced with a BLSTM layer.

Among the methods listed above, we used the CNN and BLSTM algorithms to model the temporal data and the remaining algorithms for the aggregated and temporal-quarterly data. Deep learning (CNN and BLSTM) was the feasible choice to model the temporal-monthly data owing to its ability to handle large data inputs through GPU parallelization, while the number of features in temporal data was too large for the other algorithms. Conversely, the aggregate data was not suitable for the deep learning approaches since they are explicitly designed for two-dimensional or temporal data.

To prevent any bias in our models from the skewed class distribution, we assigned weights to the following classes: 4 to the minority class (CKD) and 1 to the majority class (non-CKD). The Logistic Regression required further data normalization. This was achieved by scaling the age by the maximum age and binarizing the diagnosis and drug prescription counts. The binarization was done by setting any non-zero values to 1. Normalization had no positive effect on the other models.

To incorporate temporal effects in the machine learning models and compare their performance with the deep learning approaches, we used the temporal-quarterly data. We used the feature importance score based on information gain from our LightGBM model (trained on the aggregated data) to select the strongest predictors. An in-depth explanation of the feature importance analysis is available in Section 3.2.

A subset of methods’ hyperparameters was tuned with 5-fold cross-validation on the training set. The test set on which the final models were evaluated was not used for tuning to obtain an unbiased evaluation of the models. The values of the hyperparameters used in the final models are listed in the Supporting Information.

## 3. Results

### 3.1. Model Evaluation

**Figure 6:**
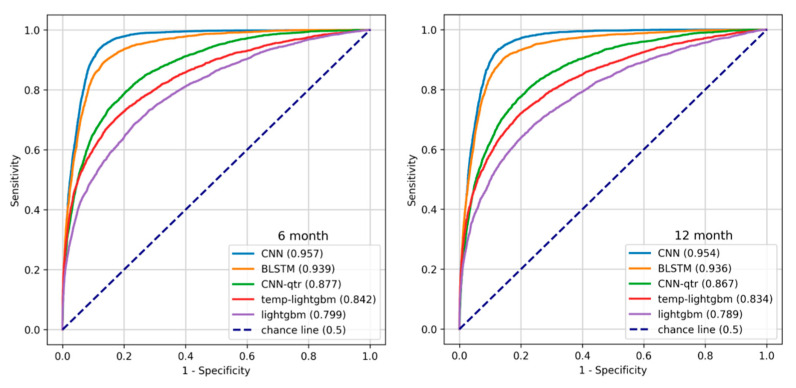
ROC curves of representative models.

We split the dataset into an 80% training set and a 20% test set. Then, we trained our models on the training set (the partition which was used for hyperparameter tuning with five-fold cross-validation) and reported their performance on the test set. The train-test and cross-validation splits were performed by stratifying using the target label. We primarily used the area under the ROC curve (AUROC) as our evaluation metric [24]. The Receiver Operating Characteristic (ROC) curve, which plots the true positive rate (Recall, Sensitivity) against the false positive rate (1—Specificity) at different probability thresholds, assesses the discriminative ability of a given binary classification model. It illustrates the model’s ability to identify positive instances while minimizing false alarms. We used Youden’s J statistic, defined as the cut-off point with the maximum difference between the True Positive Rate (TPR) and False Positive Rate (FPR), to identify the optimal probability thresholds for each model. The thresholds used for each model are tabulated in the supporting information. The plots in Figure 6 show the ROC curve for the temporal models (CNN and BLSTM), the best of temporal-quarterly with machine learning (temp-lightgbm) and deep learning (CNN-qtr) methods and aggregate (LightGBM) algorithms on 6- and 12-month data.

The confusion matrices for the CNN models are shown in Table 2. We observed that the models misclassified few positive instances and raised only a modest number of false alarms considering the larger number of negative instances. We also report accuracy, precision, recall, F1 score, and sensitivity on the CKD class for the models. Table 3 and Table 4 summarise the performance of the models on 6- and 12-month data.

To evaluate the performance of our models with comorbidities and medications independently, we ran 6-month CNN models with 64 and 32 CNN units, respectively. The comorbidity and the medication model scored 0.753 and 0.751 AUROC, respectively, indicating a strong interaction effect between the two data types.

To understand the amount of training data required to build a CNN model, we trained multiple CNN models while varying the size of the input training data and recorded the AUROC performance on our test set. Similarly, we also compared the performance of the model while varying the size of the input feature set. The features were sorted based on the importance score obtained from our LightGBM model (Discussed in Section 3.2).

**Figure 7:**
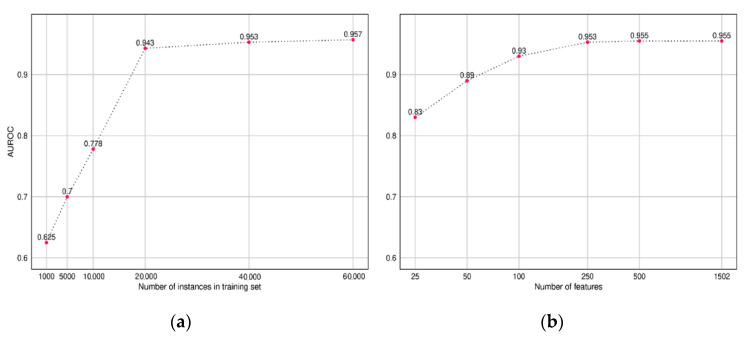
Performance of the CNN model across different sizes of (**a**) training data and (**b**) feature set.

The plot in Figure 7 presents the results, which indicate diminishing returns after 20,000 instances and 500 features, respectively.

### 3.2. Feature Importance from Boosting Methods

One of the advantages of using tree-based algorithms is that they are interpretable (unlike deep learning algorithms, which act as “black-box” with complex feature interactions and a large number of model parameters). While a human can simply look at plain decision trees, interpretation is more difficult for boosting and forest models. However, feature importance can still be derived from such models using the computed information gain values.

The information gain measures the reduction in entropy when dividing a set of data into subsets, where entropy is a measure of uncertainty. For example, if in a set of patients, the probability of having CKD is 50%, the entropy is the highest. If we could divide this set into two pure sets containing CKD and non-CKD patients, respectively, the resulting entropy is 0. For each attribute, we could measure its contribution to the reduction in entropy, i.e., its contribution to the improved explanation of the target variable. This also enabled its application in feature selection.

We plotted the feature importance for men and women to identify the key predictors associated with sex. The normalized feature importance was derived from the LightGBM models. This model interpretation primarily corroborates the reliability of the models developed from our approach by comparing it with our prior knowledge on CKD.

**Figure 8:**
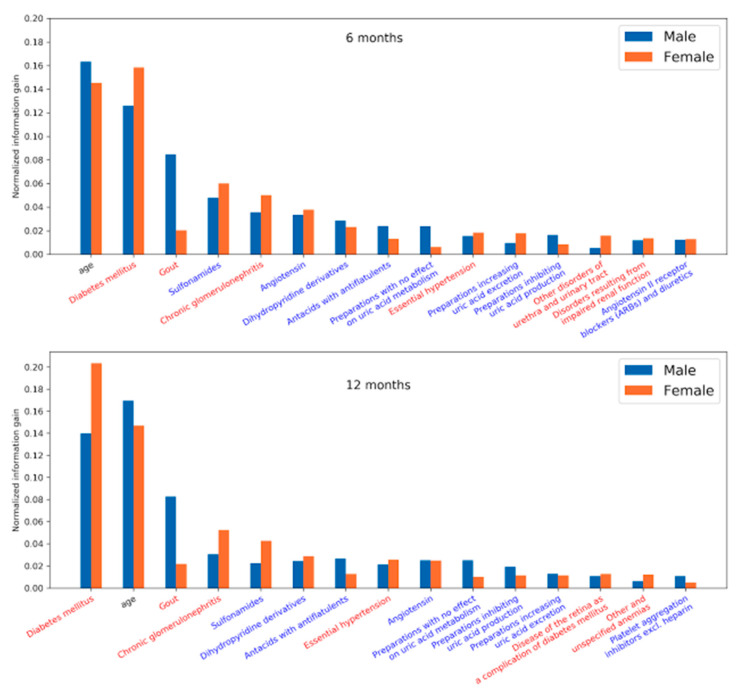
Feature importance for the LightGBM models for men and women for 6 and 12 months. The color of the x-axis labels is related to the type of feature: comorbidities are red, medications are blue, and age is black.

The results unveiled in Figure 8 highlight the factors that influence the development of CKD and were observed 6 and 12 months prior to the diagnosis of CKD. Diabetic nephropathy is a common complication of Diabetes mellitus (DM), a major precursor for CKD. In addition, medications related to DM, gout, and hypertension are seen as risk factors. Due to kidney disease, filtration of uric acid is compromised—causing gout. Gout may also lead to the progression of kidney disease.

## 4. Discussion

Among the machine-learning models, deep neural networks (CNN and BLSTM) outperformed the classical models considerably. This is interesting as they are not as well established in EHR-based health risk prediction as in some other fields (most notably computer vision). Considering that our networks were not very large or complex, the most likely reason was that they took advantage of temporal information. This is apparent from the improved performance of the classical models on the temporal-quarterly data. Introduction of temporal effects with the temporal-quarterly data to the models significantly improved the performance of the boosting methods while reducing the performance of the decision tree and logistic regression. This is in agreement with boosting models’ ability to capture complex relationships on large feature spaces.

In contrast to the commonly used approach that processes laboratory data in addition to other patient data, we developed a method that does not require laboratory data but processes only patients’ diagnoses, prescriptions, and basic demographic data (i.e., age and sex), since such data is typically available on a larger scale. This was considerably different from related studies that used laboratory values; for example, Jardim et al.—a comparison of the performances is not directly applicable.

Looking at the most prominent features in the LightGBM models, we see that age was among the strongest predictors in both the 6 and 12 month models. Among comorbidities, diabetes mellitus was the strongest predictor in both models, followed by gout, chronic glomerulonephritis, and essential hypertension. The medications most associated with the target, according to the model, were sulfonamides, followed by angiotensin, dihydropyridine derivatives, and antacids with antiflatulents. There are some differences in feature importance for men and women. While age appeared to be a slightly stronger predictor for men and diabetes mellitus for women, the most obvious difference was in gout, where the feature importance is much stronger for men than for women.

It is reasonable to see age as a strong predictor as CKD typically appears in older people. Diabetes mellitus and gout are strongly correlated with decreasing kidney function, as do chronic glomerulonephritis and essential hypertension [25]. Gout is a stronger feature in men, which could be related to different diets and lifestyles [26]. The association of various medications with CKD is due to their usage in the treatment of comorbidities. For example, dihydropyridines are vasodilators and are used to treat hypertension. Angiotensin is used for blood pressure regulation. Medications dealing with uric acid production and secretion are used to treat gout. Sulfonamides are a group of antibiotics that are used to treat urinary tract infections. Repeated infections may lead to the onset of CKD. Antacids with antiflatulents are usually prescribed to people with several other comorbidities as part of a series of medications to reduce the burden of other medications on the gastrointestinal tract. This clearly correlates with age, as older people tend to have more conditions that require polymedications, and CKD is more prevalent among the older age groups.

## 5. Limitations

Our study was built upon the data extracted from Taiwan NHI that includes age, sex, comorbidities, and medications. However, laboratory test results were not included. Therefore, our approach is appropriate for a population study but not recommended for assisting clinicians in assessing the risk for an individual patient. For clinical practice, the decisions based on laboratory tests would be more reliable. Adaptation of our method for clinical application would require further analysis and evaluation in a clinical trial. We would also need to evaluate the model on more recent data to identify potential model drift. If the model was used for decisions such as the need to set up a new dialysis center or to launch a public awareness campaign, the performance metrics we obtain are adequate. These models can also aid in proactively identifying CKD susceptible individuals in an entire region or in large groups without the need for laborious physical tests.

Since the study was carried out on the data from Taiwan, it was limited by geography and demographics. Hence, it imposes constraints on the generalizability of the model to the global population or a different region. The presence of noise in the data from human and technical errors, which are difficult to identify, may also affect the performance of the model.

## 6. Conclusions

In this study, we developed and evaluated a series of artificial intelligence-based models considering minimum variables such as sex, age, comorbidities, and medications. These models predict patients’ risk of developing chronic kidney disease after a period of 6 or 12 months. Among various models tested, convolutional neural networks (CNN) performed best, with an AUROC metric of 0.957 and 0.954 for 6 and 12 months, respectively. To see which features are the most prominent for prediction, we looked at the tree-based LightGBM model. The most prominent features included diabetes mellitus, age, gout, and use of sulfonamides and angiotensins, which are all reasonable in view of CKD.

From a policymaker’s point of view, these ML-based models could be efficiently used in resource management and initiating public health initiatives such as closely monitoring and early detection of CKD. Clearly, for the application of such models into clinical practice dealing with individual patients, the feature set would have to be expanded to include laboratory measurements and possibly lifestyle information, which falls within the scope of future work.

## Figures and Tables

**Table 1:** Characteristics of the dataset.

Predictors		
Demographics		
Age (mean ± std)	Numerical	CKD: 65 ± 15.01, Non-CKD: 68.79 ± 14.2
Gender	Categorical	Males in CKD: 57.4%, Males in Non-CKD: 57.9%
		
Diagnosis and procedures		
ICD-9 based frequencies	Numerical	Frequency of visits with a diagnosis for each of 965 ICD codes
		
Medication		
ATC-based frequencies	Numerical	Frequency of prescriptions for each of 537 ATC codes
		
**Target**		
		
CKD	Categorical	20% diagnosed with CKD

**Table 2:** Confusion matrices of the CNN model, showing the number of instances in each class, while the fraction of predicted instances are in parentheses.

**12-Month Model**		
	Predicted **Negative**	Predicted **Positive**
Actual **Negative**	12,841 (0.9)	1359 (0.1)
Actual **Positive**	423 (0.12)	3169 (0.88)
**6-Month Model**		
	Predicted **Negative**	Predicted **Positive**
Actual **Negative**	13,035 (0.91)	1288 (0.09)
Actual **Positive**	411 (0.11)	3186 (0.89)

**Table 3:** Performance metrics for 6-month data.

Dataset Type	Algorithm	Accuracy	F1	Precision	Recall or Sensitivity	Specificity	AUROC
Temporal-monthly	CNN	**0.89**	**0.773**	**0.657**	**0.94**	**0.877**	**0.957**
	BLSTM	0.87	0.735	0.624	0.893	0.865	0.939
Aggregated	LightGBM	0.751	0.525	0.426	0.685	0.767	0.799
	logistic	0.736	0.503	0.405	0.664	0.754	0.761
	randomforest	0.725	0.488	0.390	0.652	0.743	0.762
	decision tree	0.732	0.483	0.395	0.622	0.76	0.745
Temporal-quarterly	CNN	0.814	0.620	0.525	0.757	0.828	0.876
	BLSTM	0.801	0.602	0.503	0.749	0.814	0.860
	LightGBM	0.801	0.588	0.505	0.704	0.826	0.841
	logistic	0.710	0.475	0.373	0.653	0.724	0.748
	randomforest	0.737	0.501	0.405	0.654	0.758	0.773
	decision tree	0.750	0.471	0.41	0.555	0.799	0.731

**Table 4:** Performance metrics for 12-month data.

Dataset Type	Algorithm	Accuracy	F1	Precision	Recall or Sensitivity	Specificity	AUROC
Temporal-monthly	CNN	**0.88**	**0.767**	**0.65**	**0.934**	**0.873**	**0.954**
	BLSTM	0.865	0.731	0.61	0.903	0.856	0.936
Aggregated	LightGBM	0.759	0.524	0.437	0.654	0.786	0.789
	logistic	0.722	0.491	0.39	0.66	0.738	0.766
	randomforest	0.74	0.487	0.406	0.608	0.774	0.756
	decision tree	0.735	0.48	0.399	0.604	0.77	0.736
Temporal-quarterly	CNN	0.802	0.610	0.507	0.765	0.812	0.867
	BLSTM	0.779	0.585	0.471	0.773	0.781	0.855
	LightGBM	0.786	0.575	0.48	0.71	0.803	0.834
	logistic	0.742	0.486	0.406	0.605	0.776	0.747
	randomforest	0.741	0.495	0.41	0.627	0.77	0.758
	decision tree	0.746	0.465	0.404	0.548	0.797	0.721

## Data Availability

Dataset and the model outputs are available at https://osf.io/j3gur (accessed on 18 April 2021). The evaluation code is available at https://github.com/SuryaThiru/CKD-public (accessed on 18 April 2021).

## Supplementary Materials

The following are available online at https://www.mdpi.com/article/10.3390/healthcare9050546/s1, Figure S1 The correlation plot of all the patients (CKD and non-CKD); Figure S2 The correlation plot for the CKD patients; Figure S3 The correlation plot for the non-CKD people; Figure S4: Number of months visited by the CKD patients for diagnosis/drugs during the observation period (24 months) as a histogram; Figure S5: Number of months visited by the non-CKD patients for diagnosis/drugs during the observation period (24 months) as a histogram; Table S1. Hyperparameter values of the tested models. Models with the prefix “temp” using the temporal quarterly data; Table S2. The threshold values used for each model to compute the performance metrics.
