# Supplementary material for: Machine Learning Prediction Models for Chronic Kidney Disease Using National Health Insurance Claim Data in Taiwan
Source: Healthcare (Basel). 2021 May 7;9(5):546. doi: 10.3390/healthcare9050546 (PMC8151834; doi:10.3390/healthcare9050546)
Supplement: Supplementary file 1 [file healthcare-09-00546-s001.zip › healthcare-1173067-supplementary.pdf]

Machine Learning Prediction Models for Chronic Kidney Disease using National Health Insurance Claim Data in Taiwan

Surya Krishnamurthy, Kapeleshh KS, Erik Dovgan, Mitja Luštrek, Barbara Gradišek Piletič, Kathiravan Srinivasan, Yu-Chuan (Jack) Li, Anton Gradišek, Shabbir Syed-Abdul

Supplementary Materials

Table S1. Hyperparameter values of the tested models. Models with prefix “temp” use the temporal quarterly data.

Algorithm	Hyperparameter
Logistic Regression	solver = 'liblinear',<br>class_weight = {1:4, 0:1},<br>C = 0.01,<br>penalty = 'l1'
Decision Tree	**12 Month**<br>max_depth=8,<br>class_weight = {1:4,0:1},<br>min_samples_leaf = 30
	**6 Month**<br>max_depth=10,<br>class_weight = {1:4,0:1},<br>min_samples_leaf = 30
Random Forest	**12 Month**<br>criterion='gini',<br>class_weight = {1:4, 0:1}<br>n_estimators = 700,<br>max_depth = 18
	**6 Month**<br>criterion='gini',<br>class_weight = {1:4, 0:1}<br>n_estimators = 800,<br>max_depth = 18
LightBoost	**12 Month**<br>boosting_type = 'gbdt',

	learning_rate = 0.01,<br>n_estimators = 800,<br>scale_pos_weight=4
	**6 Month**<br>boosting_type = 'gbdt',<br>learning_rate = 0.01,<br>n_estimators = 1000,<br>scale_pos_weight=4
CNN	**Conv layer:**<br>n_filters = 64<br>filter_size = 3<br>stride = 1<br>activation = relu<br>padding = same<br><br>**Max pool layer:**<br>filter_size = 3<br>stride = 1<br><br>**Dropout:**<br>drop_probabiity = 0.4<br><br>**Dense layer:**<br>n_units = 32<br>activation = relu<br>initialization = random normal<br><br>**Output layer:**<br>activation: sigmoid<br><br>**Optimizer:**<br>algorithm: adadelta<br>learning rate: 0.1<br>epochs: 15
BLSTM / BLSTM-qtr	**Bi-LSTM layer:**<br>LSTM units: 64<br>activation: relu<br><br>**Dense layer:**<br>n_units = 32<br>activation = relu<br>initialization = random normal<br><br>**Output layer:**<br>activation: sigmoid<br><br>**Optimizer:**<br>algorithm: adadelta<br>learning rate: 0.1<br>epochs: 15
CNN-qtr	**Conv layer:**

	n_filters = 32<br>filter_size = 3<br>stride = 1<br>activation = relu<br>padding = same<br><br>**Max pool layer:**<br>filter_size = 3<br>stride = 1<br><br>**Dense layer:**<br>n_units = 32<br>activation = relu<br>initialization = random normal<br><br>**Output layer:**<br>activation: sigmoid<br><br>**Optimizer:**<br>algorithm: adadelta<br>learning rate: 0.1<br>epochs: 15
temp-lightgbm	**12 Month**<br>boosting_type='gbdt',<br>colsample_bytree=0.7275999182022543,<br>learning_rate=0.06467440873590191,<br>max_depth=10,<br>min_child_samples=20,<br>min_child_weight=0.001,<br>min_split_gain=0.34234014807063695,<br>n_estimators=750,<br>num_leaves=50,<br>reg_lambda=1.1,<br>scale_pos_weight=4,<br>subsample=0.8,
	**6 Month**<br>boosting_type='gbdt',<br>colsample_bytree=0.7942201755684853,<br>learning_rate=0.08416317594127293,<br>max_depth=15,<br>min_child_samples=20,<br>min_child_weight=0.001,<br>min_split_gain=0.3230893825622149,<br>n_estimators=400,<br>num_leaves=100,<br>reg_lambda=1.1,<br>scale_pos_weight=4,<br>subsample=0.7

temp-randomforest	**12 Month**<br>criterion='gini',<br>n_estimators = 800,<br>max_depth = 15,<br>class_weight = {1:4, 0:1}
	**6 Month**<br>criterion='gini',<br>n_estimators = 800,<br>max_depth = 18,<br>class_weight = {1:4, 0:1}
temp-decisiontree	**12 Month**<br>class_weight={0: 1, 1: 4},<br>criterion='gini',<br>max_depth=8,<br>min_samples_leaf=35,<br>splitter='best'
	**6 Month**<br>class_weight={0: 1, 1: 4},<br>criterion='gini',<br>max_depth=8,<br>min_samples_leaf=35,<br>splitter='best'
temp-LogisticRegression	**12 Month**<br>C=1,<br>class_weight={0: 1, 1: 4},<br>penalty='l2',<br>solver='liblinear'
	**6 Month**<br>C=0.1,<br>class_weight={0: 1, 1: 4},<br>penalty='l2',<br>solver='liblinear'

Table S2. The threshold values used for each model to computer the performance metrics.

Algo	Threshold	
	6 month	12 month
CNN	0.372	0.382
BLSTM	0.480	0.313
CNN-qrt	0.452	0.495
BLSTM-qrt	0.545	0.559
temp-lightgbm	0.436	0.429
temp-randomforest	0.436	0.470
temp-logistic	0.18	0.49
temp-decisiontree	0.484	0.486
lightgbm	0.483	0.514
randomforest	0.436	0.450
logistic	0.49	0.49
decisiontree	0.463	0.467

Dataset analysis

The following figures show the correlation plots of the top 25 features that have contributed to the occurrence of CKD. In these plots, the entries that start with an alphabet are the ATC codes for medications (eg: C03CA, C09CA, etc). The other entries that are fully numeric are the ICD9 codes for comorbidities (eg: 250, 274, etc).

- From the correlation plots, we can see that all three cases are similar. This indicates that the patient distribution in the dataset is similar among the CKD and the non-CKD groups.
- No two features are highly correlated with each other, with the highest being 0.5.
- The high correlation scores are between
    - 274 and M04AC, MO04AA, M04AB
      274 - ICD9 Code for Gout

M04 - ATC Code for Anti Gout medications

- None of the features are directly correlated to the occurrence of CKD (Figure S1), indicating the complexity of the relationship with the outcome.

Figure S1 represents the correlation plot of all the patients (CKD and non-CKD).

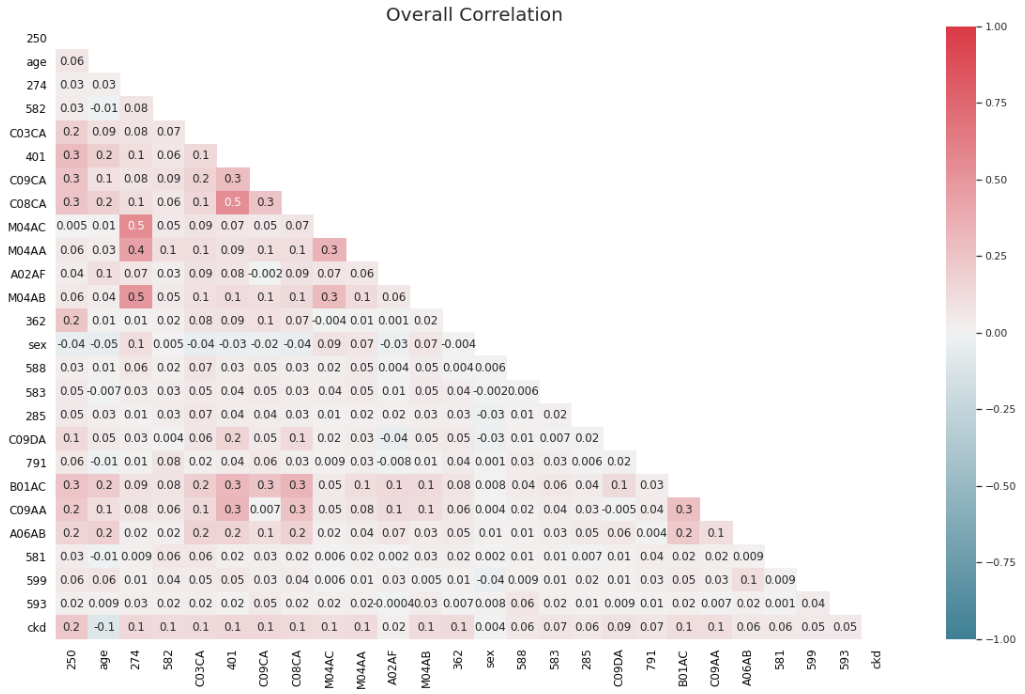

Figure S1

Figure S2 represents the correlation analysis for the CKD patients.

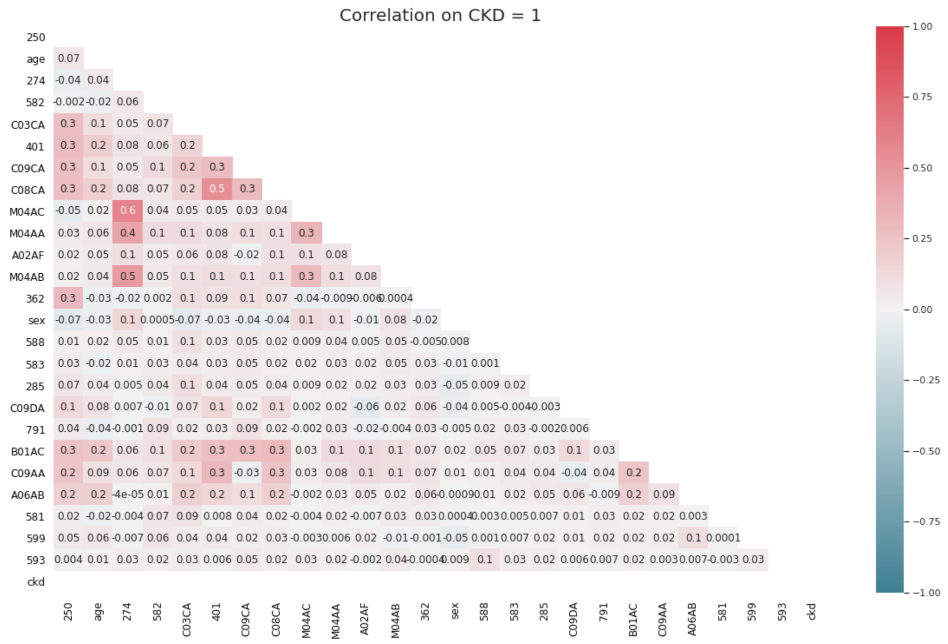

Figure S2

Figure S3 represents the correlation analysis for the non-CKD people.

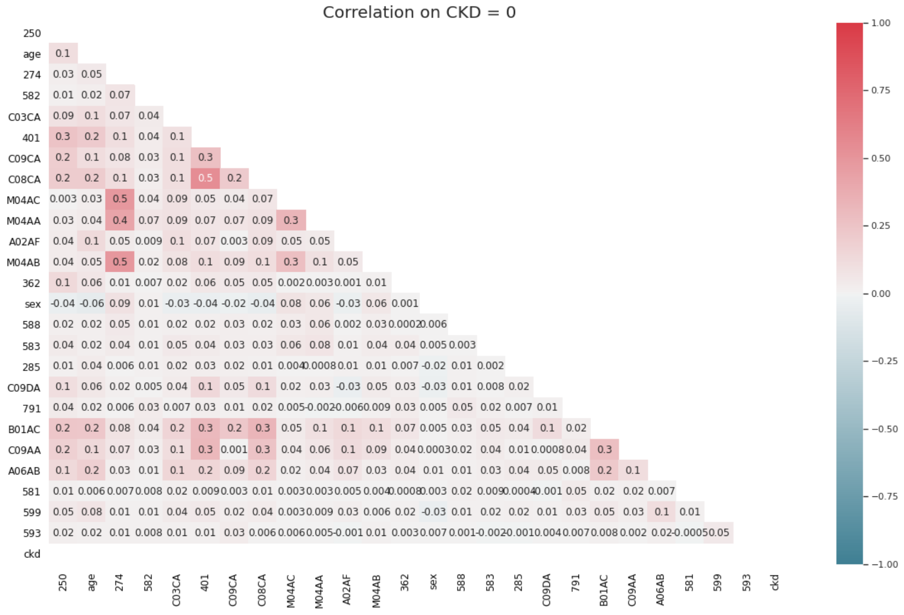

Figure S3

The following two figures show the histograms for the months visited by the patients for receiving a diagnosis or drug during the observation window in the entire dataset . Fig S4 looks at the CKD patients and Fig S5 at the non-CKD group. For example, from Fig S4 we can see that about 5800 patients visit every month (total 24 months in our observation period) for checkups and about 5000 patients visit every month to collect drugs. Both groups show similar trends (one should keep in mind that the non-CKD set is 4 times larger). We also see that most of the patients in the cohort visit every month.. .

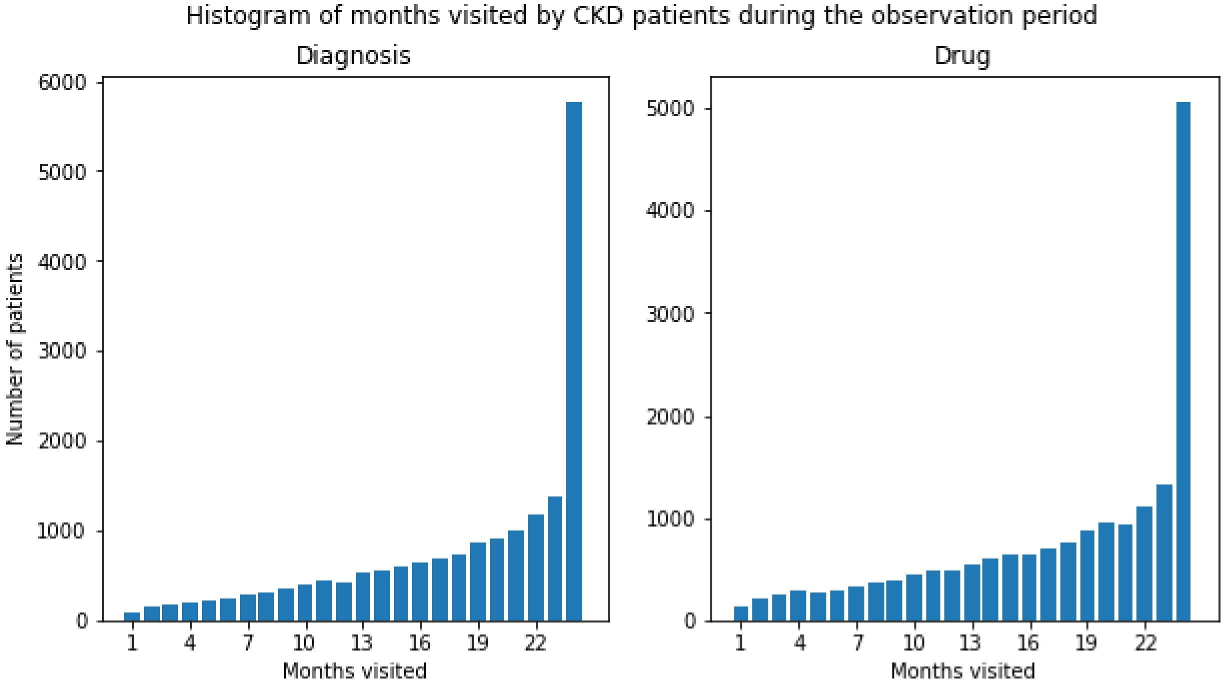

Figure S4: Number of months visited by the CKD patients for diagnosis/drugs during the observation period (24 months) as a histogram

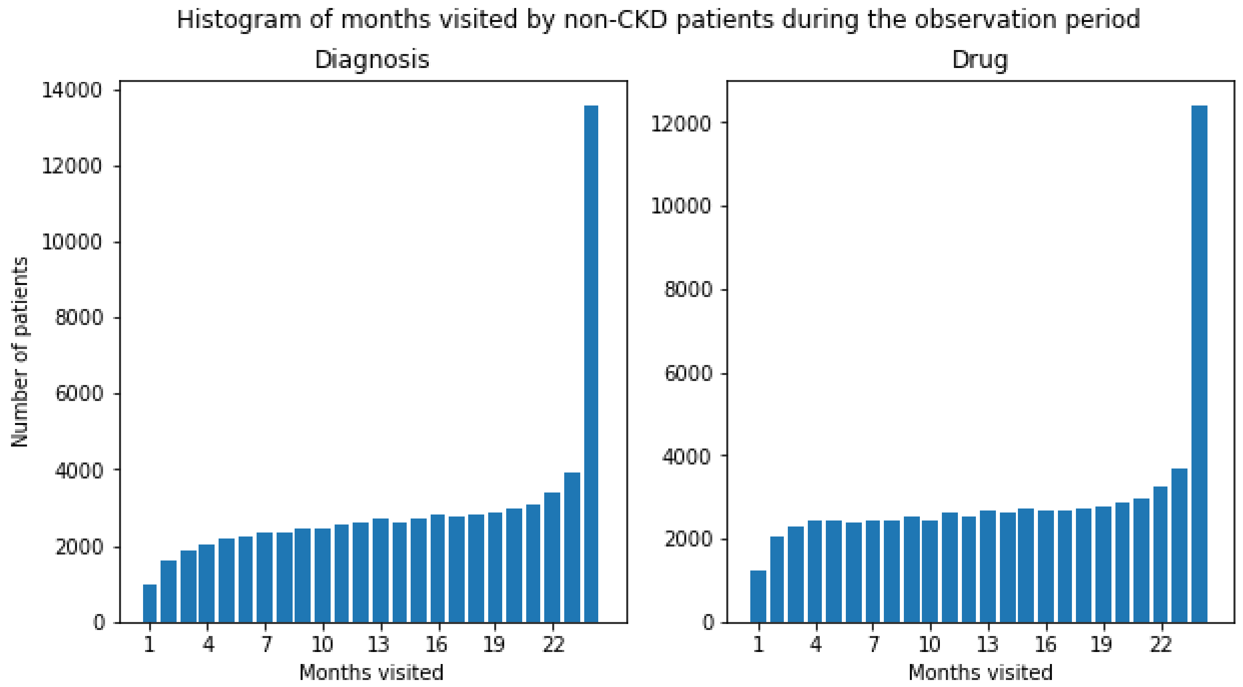

Figure S5: Number of months visited by the non-CKD patients for diagnosis/drugs during the observation period (24 months) as a histogram
